# Effect of Different Substituents on the Properties of 4-R-1,5-Diaminotetrazolium Pentazolate Salts

**DOI:** 10.3390/ma18051077

**Published:** 2025-02-27

**Authors:** Xiaofeng Yuan, Ze Xu, Ming Lu, Yuangang Xu

**Affiliations:** School of Chemistry and Chemical Engineering, Nanjing University of Science and Technology, Nanjing 210094, China

**Keywords:** nitrogen-rich compounds, tetrazolium, weak interactions, pentazolate salts, quantum chemistry

## Abstract

To explore the impact of different substituents (R) in 4-R-1,5-diaminotetrazolium cations on the performance of their pentazolate salts, five types of pentazolate salts with different groups were designed: -H, -OH, -NH_2_, -NH-NH_2_, and -N_3_. Quantum chemical methods were employed to deeply study the interionic interactions and detonation properties of these 4-R-1,5-diaminotetrazolium pentazolate salts. Among these five ionic compounds, the 1,5-diamino-4-hydroxytetrazolium pentazolate ([DAT-OH^+^] [N_5_^−^]) system exhibited the lowest interaction energy and highest stability, while the 1,5-diamino-1H-1,2,3,4-tetrazolium pentazolate ([DAT-H^+^] [N_5_^−^]) system was the least stable. Symmetry-adapted perturbation theory (SAPT) analysis indicated that electrostatic and dispersion effects predominantly contributed to these interactions. An independent gradient model based on Hirshfeld partition (IGMH) analysis further highlighted the interionic interaction regions, revealing extensive van der Waals interactions and the formation of N-H…N type hydrogen bonds. The hydrogen bond formed by the *cyclo*-N_5_^−^ and hydroxyl groups was relatively strong, while other hydrogen bonds were weaker. Benefiting from a higher enthalpy of formation, the 1,5-diamino-4-azidotetrazolium pentazolate ([DAT-N_3_^+^] [N_5_^−^]) compound exhibited the highest detonation performance (D: 9295.77 m·s^−1^; P: 32.13 GPa), while [DAT-OH^+^] [N_5_^−^] also demonstrated good performance and stability (D: 8924.96 m·s^−1^; P: 28.85 GPa).

## 1. Introduction

A renewed emphasis in the study of energetic materials has been placed on the synthesis of nitrogen-rich and polynitrogen-rich compounds in the last few years [1,2,3]. This superiority is largely due to the significantly higher energy release from the cleavage of high-energy N-N bonds compared to the energy released from redox reactions involving carbon backbones and energetic groups in traditional energetic materials. Furthermore, the final products of these reactions are often types of environmentally friendly nitrogen gas [4,5,6]. Since the discovery of N_2_, extensive research has been conducted on polynitrogen compounds, but substantial progress has been limited. It was not until 1956 that Ugi and colleagues synthesized aryl pentazole [7]. Despite this breakthrough laying the foundation for subsequent research, attempts to obtain *cyclo*-N_5_^−^ repeatedly failed due to poor structural stability, causing related studies to be halted. The synthesis of polynitrogen compounds then entered a prolonged period of stagnation. In 1999, Christe synthesized the ionic compound N_5_AsF_6_ for the first time in an anhydrous HF solvent at −78 °C. The inability of electrons in N_5_^+^ to delocalize extensively within its chain structure resulted in a decomposition temperature of only 22 °C, indicating its instability and propensity for rapid decomposition [8]. Advances in theoretical chemistry and synthesis techniques have since enabled extensive research into the synthesis pathways, decomposition mechanisms, and detonation performance of polynitrogen compounds. In 2013, Gerber et al. reported predicted results for N_8_ crystals [9]. Subsequently, Ma and colleagues successfully predicted the crystal morphology of LiN_5_ under high pressure using their independently developed CALYPSO software. When the pressure in the system was more than 9.9 GPa, LiN_5_ was determined to be thermodynamically stable [10]. That same year, Professor Haas made significant progress in the preparation of *cyclo*-N_5_^−^, achieving its synthesis by reducing phenylpentazole radicals with alkali metals and selectively cleaving C-N bonds. However, it could only remain stable at −40 °C [11]. Building on this, Zhang et al. successfully synthesized pentazolate composite ionic salt (N_5_)_6_(H_3_O)_3_(NH_4_)_4_Cl through the oxidative cleavage of *cyclo*-N_5_^−^ from aryl pentazole, and its decomposition temperature was 116.8 °C [12]. Despite ongoing debates over the structure of (N_5_)_6_(H_3_O)_3_(NH_4_)_4_Cl [13,14,15], this was the first attempt to use single-crystal X-ray diffraction to describe the solid-state structure of *cyclo*-N_5_^−^. Subsequently, Xu successfully synthesized a series of metal pentazolate salts, marking the development of the fourth polynitrogen species capable of maintaining a stable existence at room temperature and pressure [16]. This milestone opened a new chapter in the synthesis of pentazolate compounds. A series of non-metal pentazolate salts, represented by [N_2_H_5_^+^] [N_5_^−^] and [NH_3_OH^+^] [N_5_^−^], were successfully prepared [16,17,18], and the salt with the most outstanding comprehensive performance among metal pentazolate salts, LiN_5_, was introduced in 2021 [19].

To further enhance the energy of pentazolate salts, our research group attempted to synthesize diamino-pentazolium cations [20]. However, due to their poor stability, they could not be isolated. Thus, we focused on tetrazolium cations, which offer slightly less energy but greater stability. Tetrazole compounds easily undergo protonation to form stable cations, and through metathesis reactions, they can be assembled with *cyclo*-N_5_^−^ to obtain a series of energetic ionic salts. Tang et al. assembled 1,4,5-triaminotetrazolium cations with *cyclo*-N_5_^−^ to form an ionic salt, and the fact that its theoretical detonation velocity is 9487 m·s^−1^ proves the feasibility of this strategy [21]. Building on existing compounds like the 1,4,5-triaminotetrazolium cation (DAT-NH_2_^+^), 1,5-diamino-1H-1,2,3,4-tetrazolium cation (DAT-H^+^), and 1,5-diamino-4-hydroxytetrazolium cation (DAT-OH^+^), we further designed the 1,5-diamino-4-azidotetrazolium cation (DAT-N_3_^+^) and 1,5-diamino-4-hydrazinotetrazolium cation (DAT-N_2_H_3_^+^) [22,23,24]. Using quantum chemistry methods, we investigated the interactions between these five tetrazolium cations and *cyclo*-N_5_^−^ with the purpose of comprehending how various substituents impact the characteristics of 4-R-1,5-diaminotetrazolium pentazolate salts. This work provides guidance for the synthesis of 4-R-1,5-diaminotetrazolium pentazolate salts and serves as a reference for other studies on high-energy pentazolate salts.

## 2. Calculation Details

**Figure 1:**
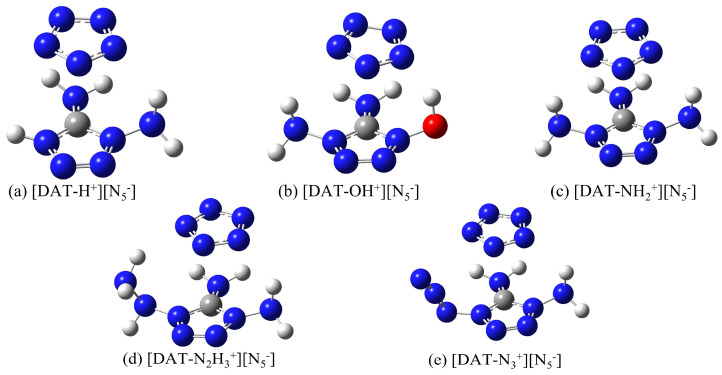
The five stable configurations after structural optimization.

To develop more stable ionic compound models, a combined approach utilizing Gaussian 16 [25] and Molclus 1.9.9.7 software [26] was employed, generating 30 configurations for each compound, which were subsequently optimized at the M062X-D3/6-311+G** level [27,28]. The high-quality integral grid of M062X excels in ring-conjugated systems; its high 54% HF exchange contribution makes it particularly effective in describing weak interactions, especially van der Waals interactions. The 6-311+G** basis set adds diffuse functions and polarization functions to heavy atoms (non-hydrogen atoms), which allows for a more accurate description of systems with widely distributed electron clouds, while the DFT-D3 correction accurately captures dispersion interactions. Moreover, dispersion corrections are equally crucial for polar ionic systems. From each set of 30 configurations, the lowest-energy, most stable structures were selected and re-optimized at the M062X-D3/ma-TZVP level, accompanied by frequency calculations [29]. This rigorous protocol ensures the reliable and precise characterization of these ionic compounds, leveraging the strengths of both methods to produce a comprehensive and accurate model. It is worth noting that the optimized configurations are all in the ground state, and the eigenvalues of the Hessian matrix are all positive, ensuring that the energy resides at a local minimum on the potential energy surface. The resulting distinct configurations of 4-R-1,5-diaminotetrazolium pentazolate salts are illustrated in Figure 1.

Subsequent to structural optimization, the interaction energy of five systems based on the ORCA 5.0.4 software package was also calculated, in which the DLPNO-CCSD(T)/ma-def2-TZVPP→QZVPP approach was applied [30,31,32,33,34,35]. Specifically, DLPNO-CCSD(T) improves upon the traditional CCSD(T) method through localized approximation techniques, reducing computational costs while maintaining high accuracy close to that of CCSD(T), particularly in energy calculations. Basis set extrapolation significantly mitigates basis set truncation errors, yielding results that are closer to the complete basis set limit. Although the computational cost of basis set extrapolation remains high for very large molecular systems, these high-precision methods are crucial for ensuring accuracy and enhancing the reliability of the calculations in this work. In addition, symmetry-adapted perturbation theory (SAPT) [36] was applied using the PSI4 software package [37] to obtain detailed information about the interaction energy components, and the calculation level was the SAPT2+(3)δMP2/aug-cc-pVTZ level [38]. Furthermore, the types, strengths, and essences of interactions within the systems were thoroughly analyzed via atom-in-molecule (AIM) theory [39], an independent gradient model based on Hirschfeld (IGMH) [40] and electrostatic potential (ESP) [41]. Ultimately, the goal of any material design and synthesis endeavor is practical application. Therefore, to identify superior-performing pentazolate salts, the detonation performance of the five systems was computed, with all thermodynamic data obtained using the G4(MP2)-6X composite method [42]. The entire analysis workflow was facilitated by the Multiwfn program developed by Lu et al. [43], and visualization was accomplished using the VMD software package [44]. These expensive yet precise computational methods are not only perfectly suited for this work but also maximize the accuracy of the computational data, further enhancing the credibility of this study. Moreover, this comprehensive, multi-faceted approach enabled a rigorous evaluation of the interplay between molecular structure, interaction mechanisms, and detonation performance, thereby informing the rational design of optimized 4-R-1,5-diaminotetrazolium pentazolate salts for practical applications.

## 3. Results and Discussion

### 3.1. Interaction Energy and Its Composition Analysis

The interaction energies between the anion and cation of each of the five 4-R-1,5-diaminotetrazolium pentazolate salts were computed, with careful correction for basis set superposition error (BSSE) using the “ghost atom” approach [45]. Specifically, $E_{A,\mathrm{AB}}$ represents the energies of the isolated anion in the presence of ghost atoms, and $E_{B,\mathrm{AB}}$ is the energy of cations in the same situation. The resulting interaction energies, *E*_int,orca_ and *E*_int,SAPT_, obtained from the ORCA and SAPT methods, respectively, are tabulated in Table 1, along with other relevant data for each system. This rigorous BSSE correction ensures accurate and reliable estimates of the interaction energies, enabling meaningful comparisons and insights into the subtle variations in interionic interactions across the series of pentazolate salts. The concurrent application of both ORCA and SAPT approaches provides robust, multi-method validation of the computed interaction energies, further solidifying the conclusions drawn from these results.

**Figure 2:**
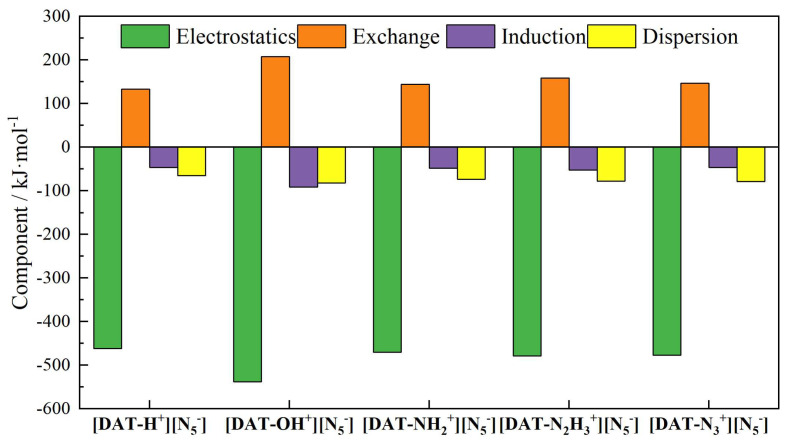
Interaction energy decomposition components of five systems.

The interaction energies among the five systems, ranked from smallest to largest, are [DAT-OH^+^] [N_5_^−^] < [DAT-N_3_^+^] [N_5_^−^] < [DAT-N_2_H_3_^+^] [N_5_^−^] < [DAT-NH_2_^+^] [N_5_^−^] < [DAT-H^+^] [N_5_^−^]. The fact that the basis set error is mostly kept under 0.3 kJ·mol^−1^ is a testament to how accurate the approach is. The close agreement between the ORCA and SAPT results validates the computational outcomes and underscores [DAT-OH^+^] [N_5_^−^] as the most stable structure among the five. The decomposition of interaction energies in Figure 2 reveals that electrostatic interactions dominate the stability of each system, facilitating attractive forces between the ions. The stronger polarity and higher electron density of the hydroxyl group compared to other substituents result in the largest absolute electrostatic interaction energy with *cyclo*-N_5_^−^, which fundamentally underlies the exceptional stability of [DAT-OH^+^] [N_5_^−^]. Moreover, the relatively large exchange–repulsion term in this system does not contradict the enhanced electrostatic attraction. In fact, even though the [DAT-OH^+^] cation is in a positive valence state as a whole, the repulsion between some electron-intensive regions and *cyclo*-N_5_^−^ is equally significant. As the substituent on the cation progresses from -H to -NH_2_ to -N_2_H_3_, the absolute values of electrostatic interactions increase. This trend likely reflects the strengthening of the hydrogen bond between the cation and *cyclo*-N_5_^−^. In the [DAT-N_3_^+^] [N_5_^−^] system, although the azido substituent lacks hydrogen atoms, its interaction energy ranks second only to the [DAT-OH^+^] [N_5_^−^] system. Calculations reveal that the atomic dipole moment-corrected Hirshfeld (ADCH) charge on the central N atom of the azido group is +0.119. This positive charge implies that, within a certain distance range, classical Coulombic interactions occur between this positively charged N atom and the negatively charged *cyclo*-N_5_^−^, enhancing the stability of the ionic system.

### 3.2. IGMH Analysis

**Figure 3:**
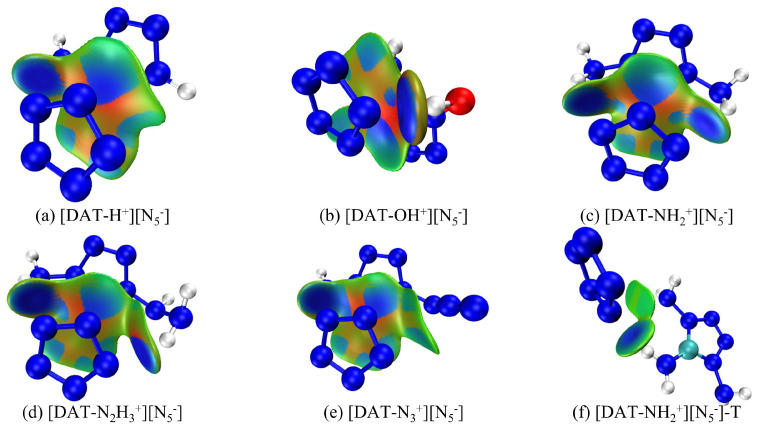
IGMH diagram of ionic systems with different substituents, (**a**–**e**) are based on their optimized structures and (**f**) is based on the work of Tang et al. [24].

IGMH provides a direct and effective means of visualizing intermolecular interactions, as it not only identifies and characterizes interaction regions but also quantitatively assesses their strengths and nature. To gain deeper insight into the ionic interactions between anions and cations in [DAT-H^+^] [N_5_^−^] and related compounds, IGMH plots for six structures are displayed in Figure 3, in which distinct colors differentiate between interaction types: repulsive, van der Waals, and attractive interactions are symbolized by the hues red, green, and blue, respectively. These plots offer a nuanced, spatially resolved representation of the intricate interplay between the electrostatic attraction, steric repulsion, and dispersion forces governing the stability and specificity of these ionic assemblies. Specifically, the IGMH method is based on Hirshfeld atomic space partitioning, which divides the actual electron density of a system. This approach not only identifies weak interactions such as hydrogen bonds but also provides a better characterization of van der Waals interactions. By explicitly mapping the characteristic interaction patterns, IGMH analysis facilitates a detailed, chemically intuitive understanding of the subtle variations in interionic bonding across this series of pentazolate salts, thereby informing the rational design of tailored ionic materials.

**Figure 4:**
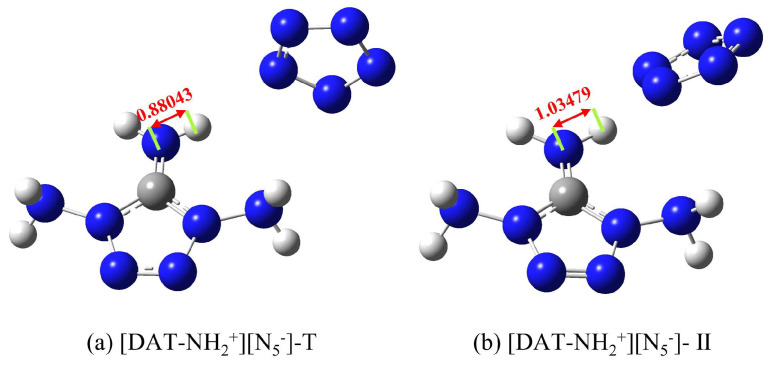
Configuration diagram of [DAT-NH_2_^+^] [N_5_^−^] isomers; (**a**) is based on experimental work by Tang et al. [24], and (**b**) is a similar configuration obtained by screening.

From Figure 3a–e, it is evident that the amino groups at the 1-position of each tetrazolium cation form hydrogen bonds with the N atoms of *cyclo*-N_5_^−^, contributing to the stability of the system. Notably, light blue regions, resembling “petal-like” features, appear on the interaction surface, suggesting a π-π stacking interaction. However, despite the overlapping arrangement of *cyclo*-N_5_^−^ with different tetrazolium cations, it lies above the C-NH_2_ region with limited overlap area. Therefore, two plausible scenarios emerge for the interaction region: (I) the H atoms on C-NH_2_ form weak hydrogen bonds with *cyclo*-N_5_^−^, or (II) the N atoms of the amino group engage in electrostatic interactions with *cyclo*-N_5_^−^. The light blue color indicates reduced electron density, possibly exceeding only van der Waals interactions. However, the IGMH method can only find out the range of interaction between them but cannot judge the type of interaction. Furthermore, the deepest blue color at the hydrogen bond site between the hydroxyl group and *cyclo*-N_5_^−^ in [DAT-OH^+^] [N_5_^−^] signifies high electron density and a strong hydrogen bond, while the most intense red regions, also in [DAT-OH^+^] [N_5_^−^], indicate pronounced repulsive interactions, consistent with SAPT analysis. Interestingly, Tang et al. reported a distinct triaminotetrazolium pentazolate salt structure (Figure 4a), differing from our constructed models. A similar structure was obtained during configurational searching (Figure 4b), but with higher energy and inferior structural quality, suggesting that Tang’s structure likely exhibits poor stability. To further validate this hypothesis, the interaction energy of [DAT-NH_2_^+^] [N_5_^−^]-T was calculated. After accounting for the BSSE effect, the calculated interaction energy was −325.07 kJ·mol^−1^, with a total energy of −1,831,831.71 kJ·mol^−1^. These values are both higher than those of the overlapping structure, clearly indicating the inferior stability of the structure in Figure 4a. Furthermore, literature reports of extensive hydrogen bonds in triaminotetrazolium pentazolate crystals are indeed correct, with many H atoms in the amino groups forming bonds with electro-negative *cyclo*-N_5_^−^. However, the light blue regions in Figure 3f reveal extremely weak hydrogen bonds. The side-by-side structure of the anion and cation reduces the van der Waals interaction area, which is not conducive to the stability of the whole system. Notably, the N-H bond length of 1.035 Å in Figure 3f exceeds the 0.880 Å value in the original crystal, implying that *cyclo*-N_5_^−^ polarity is reduced under hydrogen bond constraints. Due to the constraints of various weak interactions, including hydrogen bonds, within the crystal lattice, the length of this N-H bond is only 0.880 Å. However, in an isolated system—that is, when the ionic salt geometry is optimized and freed from the constraints of hydrogen bonds and other interactions—the bond length increases to 1.035 Å, which is unfavorable. Upon external perturbation, structural changes within the crystal could trigger hydrogen transfer reactions, with liberated *cyclo*-N_5_^−^ potentially abstracting reactive H atoms from the cation to form HN_5_. This instability, shared by many pentazolate salts, critically contributes to their reduced stability (Figure 4a shows an initial decomposition temperature of 96.3 °C). In their work, Zhu et al. [46] discovered that [NH_3_OH^+^] [N_5_^−^] first undergoes a hydrogen transfer reaction to form HN_5_ during decomposition. Furthermore, experimental studies by Li et al. [47] demonstrated that the decomposition temperature and activation energy of [N_2_H_5_^+^] [N_5_^−^] are both related to hydrogen bonds. This not only validates the correctness of this work but also demonstrates the value of computational simulations in design and synthesis. Crucially, this work reveals that at least one triaminotetrazolium pentazolate isomer with a more stable overlapping structure may exist, underscoring the importance of exploring alternative configurations to optimize stability.

### 3.3. AIM Analysis

**Figure 5:**
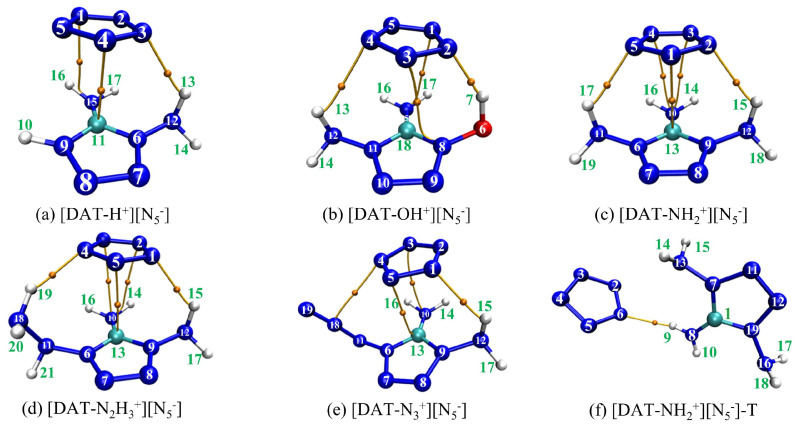
Topological diagram of electron density of different ion systems.

Using AIM theory as a foundation, topological analysis of electron density distributions is a crucial approach for elucidating molecular bonding characteristics. Based on Bader’s theoretical definition [48], the bond critical point (BCP) has garnered widespread attention due to its prevalence between interacting atoms and ability to reflect the nature of the interactions. Topological plots for the five potential ionic systems are presented in Figure 5, with key information succinctly summarized in Table 2. These plots provide a detailed, visual representation of the electron density landscape, enabling the identification of BCPs that unequivocally signify chemical bonding. The topological metrics associated with these BCPs can provide quantitative insights into bond strength, polarity, and π-character. By systematically analyzing these topological features, valuable information can be gleaned regarding the intricate interplay of electrostatic, covalent, and dispersion forces governing the stability and reactivity of these ionic assemblies. The integration of AIM-based topological analysis with other computational and experimental approaches thus constitutes a powerful framework for elucidating the complex relationships between molecular structure, bonding, and functionality.

For the relevant data, the potential energy density (*V*(*r*)) is typically negative, while the kinetic energy density (*G*(*r*)) is generally positive. The total energy density (*H*(*r*)), being the sum of these two, reflects the overall energy distribution at the BCP. When it comes to the critical point of a hydrogen bond, the electron density should normally be between 0.002 and 0.035 a.u., according to Popelier [49]. Building on this, Rozas proposed that a weak hydrogen bond exists when both ∇^2^*ρ* and *H*(*r*) are positive. The strength of the hydrogen bond is moderate if ∇^2^*ρ* is positive and *H*(*r*) is negative. It is said that the hydrogen bond is strong when both values are negative [50]. Data analysis reveals that all hydrogen bonds formed between the H atoms on the amino groups of the tetrazolium cation and *cyclo*-N_5_^−^ are relatively weak, with the exception of the hydrogen bond between the N2 and H7 atoms in the [DAT-OH^+^] [N_5_^−^] system, which exhibits the highest bond strength. This is a key factor contributing to the relatively low interaction energy within the [DAT-OH^+^] [N_5_^−^] system. Additionally, topological analysis of the electron density accurately identified the interaction sites between the cations and anions. Notably, *cyclo*-N_5_^−^ does not form hydrogen bonds with the H atoms in the C-NH_2_ groups, and there is minimal electron density convergence in this region. However, bond critical points are observed between N atoms and the *cyclo*-N_5_^−^ anion, which is analogous to anion–π stacking interactions. For instance, in the [DAT-NH_2_^+^] [N_5_^−^] system, the negatively charged N10 atom has an ADCH charge of −0.325, and the π-electron cloud of the *cyclo*-N_5_^−^ anion also exhibits high electron density. Under the influence of electrostatic and induction effects, the electron clouds between these ions are mutually attracted and polarized, thereby strengthening the interaction between the cation and *cyclo*-N_5_. The interaction between C atoms and *cyclo*-N_5_^−^ is similar to cation–π interactions, also governed by non-covalent interactions such as electrostatic and dispersion forces.

It is worth noting that in the [DAT-N_3_^+^] [N_5_^−^] system, the interaction between the N18 atom in the azido group and the anion is similar to that of the C atom. Although the nature of the atomic charges leads to variations in interaction mechanisms, these weak interactions collectively contribute to the stability of the system. To assess the strength of these interactions, Lu et al. suggested that a higher electron density at the BCP corresponds to stronger interactions [51]. Most of the BCPs associated with N-N and C-N bonds exhibit electron densities lower than those observed in hydrogen bonds. For example, in the [DAT-H^+^] [N_5_^−^] system, the electron density for the N3…H13 interaction is 0.01867, whereas for the N15…N1 interaction, it is only 0.01341. Among all the systems studied, the N2…H7 interaction in [DAT-OH^+^] [N_5_^−^] has the highest electron density at 0.05507, consistent with the earlier analysis. The interaction sites identified by the IGMH are largely consistent with those displayed in the electron density topology maps shown in Figure 5. However, in the [DAT-N_3_^+^] [N_5_^−^] system, the BCP between N2 and N10 could not be located, and similar issues were observed in other systems. It is important to mention that, compared to IGMH analysis, some key BCPs might not be reflected in the AIM analysis due to limitations inherent to AIM theory [52]. Finally, the electron density topology analysis of the [DAT-NH_2_^+^] [N_5_^−^]-T system indicates that the hydrogen bonds formed in the parallel structure are relatively weak, with electron densities similar to those of hydrogen bonds in other structures, showing no significant differences. This finding is in line with the results obtained from IGMH analysis.

### 3.4. Electrostatic Potential Analysis

**Figure 6:**
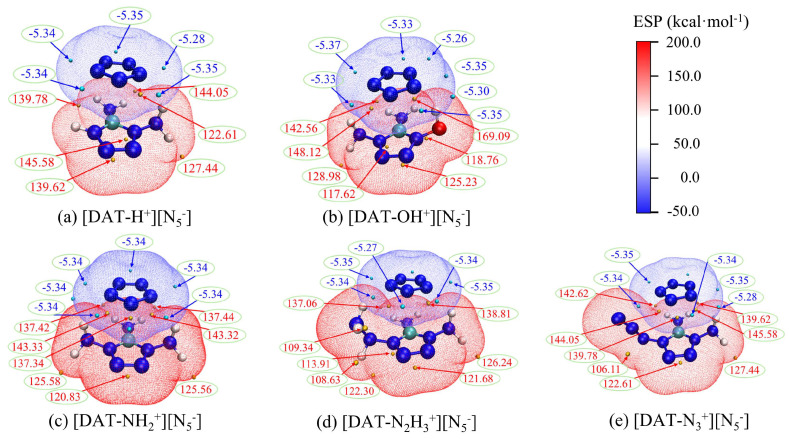
The penetration diagram of electrostatic potential between ions in composite systems.

Electrostatic potential, by describing the charge distribution across different regions of a molecule, reveals important properties such as intermolecular interactions and reactive sites. To analyze the changes occurring after *cyclo*-N_5_^−^ assembles into ionic compounds with five cations, a penetration diagram of the optimized 4-R-1,5-diaminotetrazolium pentazolate salt systems is shown in Figure 6, showing the interactions between anions and cations. This study utilizes Bader’s approach, adopting an electron density isosurface of 0.001 a.u. as the van der Waals surface [53]. In the figure, red regions indicate positive potential, while blue regions indicate negative potential, with deeper colors signifying higher absolute values of the electrostatic potential in those areas. The blue sphere represents the local minimum on the isosurface, while the orange sphere represents the local maximum.

Figure 6 reveals that the distributions of the minima of the electrostatic potential for the *cyclo*-N_5_^−^ anion are similar, with values generally around −5.30. Different substituents on the cation, however, drastically alter the distribution of positive potential maxima. Notably, the hydroxyl-substituted cation [DAT-OH^+^] exhibits a significantly higher positive potential value compared to the other four systems. This suggests a stronger polarity for the [DAT-OH^+^] cation. The majority of the positive potential maxima are located near the hydrogen atom, probably because of the existence of the less-electron-dense, positively charged hydrogen atom. Furthermore, the presence of N18 exhibiting positive charge in the [DAT-N_3_^+^] [N_5_^−^] system also contributes to the observed positive potential maxima near the N atoms.

The remaining three cation systems exhibit diverse distributions of positive potential maxima. Compared to the [DAT-H^+^] cation, the [DAT-NH_2_^+^] and [DAT-N_2_H_3_^+^] cations show a decrease in polarity. This observation is supported by the observed differences in the positions and magnitudes of the positive potential maxima. For instance, the [DAT-H^+^] system exhibits a maximum value of 139.62 at the C atom, while the other two systems show values of 120.83 and 121.68, respectively. This difference could be attributed to the presence of amino and hydrazine groups, which act as electron-donating groups, altering the electron density and distribution within the system, leading to a decrease in some positive potential maxima.

**Figure 7:**
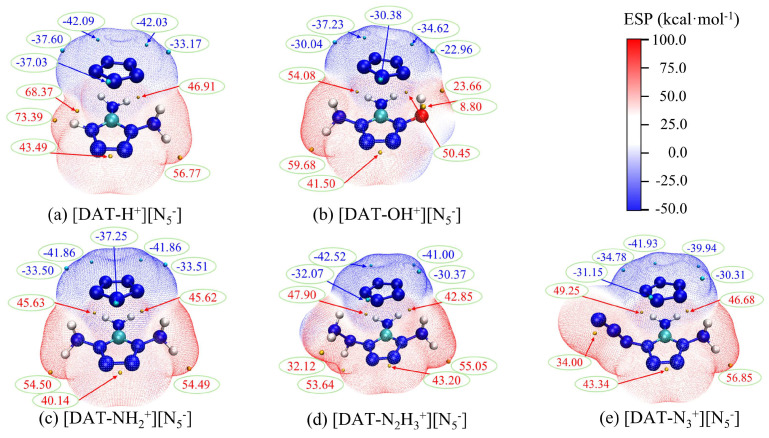
The overall electrostatic potential display of composite systems.

Furthermore, Figure 6 clearly demonstrates the penetration of the positive and negative electrostatic potential regions, highlighting the complementary nature of electrostatic potential and the fundamental role of weak interactions like hydrogen bonds. A comprehensive electrostatic potential map of the five ion systems is shown in Figure 7. The combination of the ions leads to a decrease in the polarity of the cation, and the negative potential minima of *cyclo*-N_5_^−^ are significantly reduced. Under electrostatic influence, the local electron density of the cation’s surface increases, leading to a decrease in the positive potential maxima. However, this process is accompanied by exchange and induction interactions, leading to electron redistribution and polarization between the ions. This redistribution of charge is a key factor in the enhancement of the *cyclo*-N_5_^−^ polarity.

Comparing different substituent systems reveals that the [DAT-H^+^] [N_5_^−^] system exhibits a higher positive potential maximum value, particularly near the hydrogen atom, with a maximum value of 73.39. This suggests that this region could be a preferred target for nucleophilic attack. Indeed, based on similar reaction mechanism studies [15], hydrogen transfer reactions are also likely to be the initial decomposition step in the [DAT-H^+^] [N_5_^−^] system. In the [DAT-OH^+^] [N_5_^−^] system, most of the extreme points have relatively small absolute values, indicating that the reactivity sites have low polarity, which disfavors attacks by electrophilic and nucleophilic reagents, thereby enhancing the system’s chemical stability. The remaining three systems exhibit similar extreme point locations and close values, consistent with the situation of interaction energies.

### 3.5. Detonation Performance

The detonation performance is a critical criterion for evaluating the effectiveness of energetic materials, which underlines the fundamental pursuit of high nitrogen content. As ionic compounds consist of both cations and anions, Politzer proposed an equation to calculate the density of ionic compounds [54], as shown in Equations (1) and (2). However, densities calculated without considering weak interactions are significantly erroneous. Building on Rice’s work [55], Politizer considered the volume correction and obtained a more suitable density equation, as given in Equation (3), for ionic compounds [56]. Moreover, the calculation of the enthalpy of formation of ionic compounds is quite unique. Jenkins argued that the results obtained from isolated systems do not represent crystal data accurately, and the necessity of correcting lattice enthalpy is also considered [57,58]. The method for calculating the enthalpy of formation of energetic ionic salts is shown in Equations (4)–(6).

(1) $$V_{\mathrm{uncorrected}}=pV_{M^{+}}+qV_{X^{-}}$$

(2) $$\rho (\mathrm{crystal})\;=\alpha (M/V_{\mathrm{corrected}})+\beta (V_{s^{+}}/A_{s^{+}})+\gamma (V_{s^{-}}/A_{s^{-}})+\delta$$

(3) $$V_{\mathrm{corrected}}=V_{\mathrm{uncorrected}}-[0.6763+0.9418N]$$

(4) $${∆H}_{f}\left(salt,\;298\;K\right)=∆H_{f}\left(cation,\;298\;K\right)+∆H_{f}\left(anion,\;298\;K\right)-∆H_{L}$$

(5) $$∆H_{L}=U_{POT}+[p(n_{M}/2-2)+q(n_{X}/2-2)]RT$$

(6) $$U_{POT}\left[\mathrm{kJ}{\mathrm{mol}}^{-1}\right]=\gamma {\left(\rho_{m}/M_{m}\right)}^{1/3}+\delta$$

The detonation parameters for five systems were calculated using EXPLO5 V6.05.04 software [59,60], and the configurations obtained by Tang et al. were analyzed [24], and all key information is presented in Table 3. The data from the table indicate a positive correlation between the enthalpy of formation of the systems and their nitrogen content; that is, the higher the nitrogen content, the greater the enthalpy of formation. Thanks to its exceptionally high enthalpy of formation, the [DAT-N_3_^+^] [N_5_^−^] system achieved a theoretical detonation velocity of 9295.77 m·s^−1^. Moreover, the order of densities among the systems is also remarkable, with the [DAT-N_3_^+^] [N_5_^−^] system having the highest density, likely due to the larger mass of the ions. However, a larger ionic mass is not always advantageous, as the fixed van der Waals volume of the atoms and the steric effects between ions cause the overall volume of the ionic compound to increase with the number of atoms, which is detrimental to density. To illustrate this point, although the mass of the [DAT-N_2_H_3_^+^] [N_5_^−^] ion is 201.158, greater than that of the [DAT-NH_2_^+^] [N_5_^−^] system, its corrected volume is 221.5977, close to that of the [DAT-N_3_^+^] [N_5_^−^] system, which is the fundamental reason for its lowest density. Due to its smaller ionic volume, the density of the [DAT-H^+^] [N_5_^−^] system is moderate. The [DAT-OH^+^] [N_5_^−^] system, by virtue of electrostatic attractions and other interactions compressing its volume, has a density just below that of the [DAT-N_3_^+^] [N_5_^−^] system. In particular, the [DAT-NH_2_^+^] [N_5_^−^] system warrants detailed explanation. First, the calculations indicate that the overlapped [DAT-NH_2_^+^] [N_5_^−^] system has a higher density than the experimentally obtained one (even though the values are very close), primarily due to differences in volume caused by varying numbers of hydrogen bonds, yet this is just the tip of the iceberg in their differences. Indeed, Politize’s consideration of weak interactions between ionic systems is far from adequate for this work. Even disregarding the electrostatic and induced interactions between the cation and *cyclo*-N_5_^−^, the van der Waals interactions alone would result in a smaller calculated ionic volume for the overlapped system, which is not reflected in the equations. However, it is unquestionable that the overlapped [DAT-NH_2_^+^] [N_5_^−^] system would exhibit a higher crystal density and greater stability.

## 4. Conclusions

Different R-group-substituted 4-R-1,5-diaminotetrazolium cations were designed and assembled with *cyclo*-N_5_^−^ anions to form pentazolate salts. Based on quantum chemical calculations, the minimum energy configurations of five energetic ionic salts were obtained. The interaction energies between the cations and anions were computed, and the SAPT method was used to further analyze and explain the energy components.

The interaction energies of the five ionic salts, in ascending order, are as follows: [DAT-OH^+^] [N_5_^−^] < [DAT-N_3_^+^] [N_5_^−^] < [DAT-N_2_H_3_^+^] [N_5_^−^] < [DAT-NH_2_^+^] [N_5_^−^] < [DAT-H^+^] [N_5_^−^]. Among these, [DAT-OH^+^] [N_5_^−^] exhibits the greatest stability. IGMH analysis revealed extensive van der Waals interactions and the formation of hydrogen bonds between the cations and anions in each salt. The varying substituents led to differences in the interaction strengths between the cations and *cyclo*-N_5_^−^. The AIM and IGMH analyses corroborated each other and characterized the strengths of various weak interactions. Notably, the hydroxyl group in [DAT-OH^+^] [N_5_^−^] formed the strongest hydrogen bond with *cyclo*-N_5_^−^, while all other hydrogen bonds were weaker. The N atoms of the amino groups on different cations also displayed electrostatic attraction and other weak interactions akin to anion–π interactions with *cyclo*-N_5_^−^. The electron topological analysis of the experimentally obtained [DAT-NH_2_^+^] [N_5_^−^] indicated that the hydrogen bonds formed between *cyclo*-N_5_^−^ and the H atoms of the triamino group were weak hydrogen bonds. This weakness is a crucial factor contributing to its lower decomposition temperature. Furthermore, the electrostatic potential analysis demonstrated that the [DAT-OH^+^] cation possessed the strongest polarity. Electrostatic and inductive interactions resulted in the smallest absolute values of the electrostatic potential extrema for its corresponding pentazolate salts. Consequently, the reaction sites are less susceptible to attack by electrophilic and nucleophilic reagents, enhancing the overall chemical stability. Finally, the detonation properties of the five systems were calculated. [DAT-N_3_^+^] [N_5_^−^], due to its high-energy azido group, exhibited superior detonation performance, while [DAT-OH^+^] [N_5_^−^] was slightly inferior. This theoretical work demonstrates the potential application value of the designed compounds by investigating the relationship between molecular structure and properties. The computational results will provide valuable references for experiments, reducing trial-and-error costs in future work and offering new insights into molecular design and theoretical guidance for upcoming experimental research.

## Figures and Tables

**Table 1:** Interaction energy of pentazolate salt systems with different substituents.

**System**	***E*** **_A,AB_**	***E*** **_B,AB_**	*E*_BSSE_	*E*_int,orca_	*E*_int,SAPT_
[DAT-H^+^] [N_5_^−^]	−718,006.26	−968,572.03	0.19	−438.76	−440.03
[DAT-OH^+^] [N_5_^−^]	−718,005.30	−1,165,736.11	0.05	−500.47	−502.98
[DAT-NH_2_^+^] [N_5_^−^]	−718,005.96	−1,113,698.28	0.28	−445.44	−447.86
[DAT-N_2_H_3_^+^] [N_5_^−^]	−718,005.97	−1,258,809.31	0.01	−447.63	−450.16
[DAT-N_3_^+^] [N_5_^−^]	−718,006.07	−1,397,493.97	0.09	−451.58	−454.64

Note: the units of all variables in the table are kJ·mol^−1^.

**Table 2:** The details of the topological analysis of the electron density of each ion system.

System	Interaction	*ρ*(r)	▽^2^*ρ*(r)	*H*(r)	*G*(r)	*V*(r)
[DAT-H^+^] [N_5_^−^]	N15-N1	0.01341	0.05362	0.00201	0.01140	−0.00939
	C11-N4	0.01565	0.05842	0.00161	0.01299	−0.01138
	N3-H13	0.01867	0.06528	0.00201	0.01431	−0.01229
[DAT-OH^+^] [N_5_^−^]	H13-N4	0.00868	0.03168	0.00152	0.00640	−0.00488
	N2-H7	0.05507	0.08633	−0.01455	0.03613	−0.05068
	N15-N1	0.01125	0.04504	0.00182	0.00944	−0.00763
	N3-N8	0.01616	0.06229	0.00162	0.01395	−0.01233
[DAT-NH_2_^+^] [N_5_^−^]	H15-N2	0.01728	0.06135	0.00207	0.01327	−0.01120
	N5-H17	0.01729	0.06139	0.00207	0.01328	−0.01121
	N3-N10	0.01061	0.04355	0.00177	0.00912	−0.00735
	N10-N4	0.01061	0.04355	0.00177	0.00912	−0.00735
	C13-N1	0.01425	0.05460	0.00161	0.01204	−0.01042
[DAT-NH_2_^+^] [N_5_^−^]-T	N6-H9	0.01837	0.06691	0.00260	0.01412	−0.01152
[DAT-N_2_H_3_^+^] [N_5_^−^]	H19-N4	0.01978	0.06938	0.00214	0.01521	−0.01307
	N1-H15	0.02067	0.07257	0.00195	0.01619	−0.01424
	N3-N10	0.01005	0.04271	0.00179	0.00888	−0.00709
	N10-N2	0.01060	0.04288	0.00188	0.00883	−0.00695
	N5-C13	0.01424	0.05277	0.00154	0.01165	−0.01010
[DAT-N_3_^+^] [N_5_^−^]	H15-N1	0.01740	0.06282	0.00208	0.01363	−0.01155
	C13-N5	0.01556	0.05714	0.00148	0.01280	−0.01132
	N10-N3	0.01106	0.04689	0.00193	0.00980	−0.00787
	N18-N4	0.00954	0.04171	0.00184	0.00859	−0.00675

**Table 3:** The theoretical detonation parameters of ion systems.

System	Nitrogen Content/%	*ρ* (g·cm^−3^) *^a^*	∆*H*_f_ (kJ·mol^−1^) *^b^*	*D* (m·s^−1^) *^c^*	*P* (GPa) *^d^*
[DAT-OH^+^] [N_5_^−^]	82.35	1.638	615.56	8924.96	28.85
[DAT-H^+^] [N_5_^−^]	90.06	1.590	669.06	8835.06	27.39
[DAT-NH_2_^+^] [N_5_^−^]-T	90.32	1.575	763.77	8977.92	28.16
[DAT-NH_2_^+^] [N_5_^−^]	90.32	1.583	763.15	9018.09	28.56
[DAT-N_2_H_3_^+^] [N_5_^−^]	90.55	1.569	888.32	9179.52	29.70
[DAT-N_3_^+^] [N_5_^−^]	92.45	1.643	1153.71	9295.77	32.13

*^a^* Calculated density. *^b^* Calculated heat of formation. *^c^* Detonation velocity, values calculated using EXPLO5 V6.05. *^d^* Detonation pressure, values calculated using EXPLO5 V6.05.

## Data Availability

The original contributions presented in the study are included in the article; further inquiries can be directed to the corresponding author.
